# Effect of SNPs on Creatine Kinase Structure and Function: Identifying Potential Molecular Mechanisms for Possible Creatine Kinase Deficiency Diseases

**DOI:** 10.1371/journal.pone.0045949

**Published:** 2012-09-25

**Authors:** Chang Li, Qian Zhang, Wei-Jiang Hu, Hang Mu, Zong Lin, Long Ma, Yong-Doo Park, Hai-Meng Zhou

**Affiliations:** 1 Protein Science Laboratory of the Ministry of Education, School of Life Sciences, Tsinghua University, Beijing, P.R. China; 2 School of Basic Medical Sciences, Beijing University of Chinese Medicine, Beijing, P.R. China; 3 Zhejiang Provincial Key Laboratory of Applied Enzymology, Yangtze Delta Region Institute of Tsinghua University, Jiaxing, P.R. China; 4 Bejing Key Laboratory of Protein Therapeutics, School of Life Sciences, Tsinghua University, Beijing, P.R. China; Institute of Molecular Genetics IMG-CNR, Italy

## Abstract

Single-nucleotide polymorphisms (SNPs) are common genetic material changes that often occur naturally. SNPs can cause amino acid replacements that may lead to severe diseases, such as the well-known sickle-cell anemia. We constructed eight SNP mutants of human brain-type creatine kinase (CKB) based on bioinformatics predictions. The biochemical and biophysical characteristics of these SNP mutants were determined and compared to those of the wild-type creatine kinase to explore the potential molecular mechanisms of possible creatine kinase SNP-induced diseases. While the reactivation of six SNP mutants after heat shock dropped more than 45%, only three of them showed notable increases in ANS fluorescence intensity and decreases in catalytic efficiency. Among them, H26Y and P36T bind substrates as well as the wild-type form does, but the melting temperatures (T_m_) dropped below body temperature, while the T59I mutant exhibited decreased catalytic activity that was most likely due to the much reduced binding affinity of this mutant for substrates. These findings indicate that SNPs such as H26Y, P36T and T59I have the potential to induce genetic diseases by different mechanisms.

## Introduction

Single-nucleotide polymorphisms (SNPs) are single base pair alterations of alleles either in or between individuals. The development of new techniques for the large-scale identification of SNPs in the human genome [1] has facilitated the exponential expansion of the known incidence of SNPs. According to the NIH SNP database (http://www.ncbi.nlm.nih.gov/SNP, build 130, 2009), the validated SNP cases number 7.0 million compared to 2.8 million in the year 2001. The major goal of mining this database is to find the relevance of these genetic variations and genotypes, thus providing a basis for the mechanisms of and therapies for human diseases [2]. Non-synonymous SNPs in coding regions result in the change of amino acids in proteins, which may cause structural alterations of functional proteins, leading to a loss of activity, stability, solubility or other functions. There are many diseases associated with missense SNPs, such as the well-known sickle-cell anemia [3], [4]; rheumatoid arthritis, which is caused by the dysfunction of the protein tyrosine phosphatase [5]; Li-Fraumeni syndrome [6] and congenital cataract-microcornea syndrome [7].

In vertebrates, the only existing phosphagen kinase is creatine kinase (CK, EC 2.7.3.2), which catalyzes the reversible transfer of a phosphate group between ATP and creatine [8]. It plays an important role in intracellular energy metabolism and is distributed mainly in excitable tissues, such as muscle, brain and electrogenic organs [9], [10]. There are at least five types of CK isoenzymes in vertebrates: homo-dimeric CKB (Brain type), CKM (Muscle type), hetero-dimeric MB-CK (Heart type) and two mitochondrial CKs that exist as homo-octamers [11], [12]. The most important physiological function of CK is to regenerate ATP from phosphocreatine (PCr), thus providing sufficient ATP for highly energy-demanding sites. CKB can interact with the neuron-specific K^+^-Cl^−^ co-transporter KCC2, and the microenvironmental ATP concentration, altered by CK, might be essential for the activation of this transporter [13], [14]. The activation of this transporter is accompanied by an alteration of the neuron potential, so it is reasonable to guess that CKB may also be involved in neuron transduction.

The important physiological function of CKs causes them to have many clinical applications. Dysfunctions in CKB have been observed in many diseases. CKB activity is reduced in brain regions affected by neurodegeneration in Pick’s disease, Alzheimer’s disease and Lewy body dementia [15], [16], [17]. The decrease of CK activity is not due to the down-regulation of gene expression but to the oxidative modification of CK that is induced by environmental stress [18]. Moreover, the serum CKB level is often used as a clinical biochemical marker for many diseases such as cancer [19], cerebral disorder [20] and renal diseases [21].

**Figure 1:**
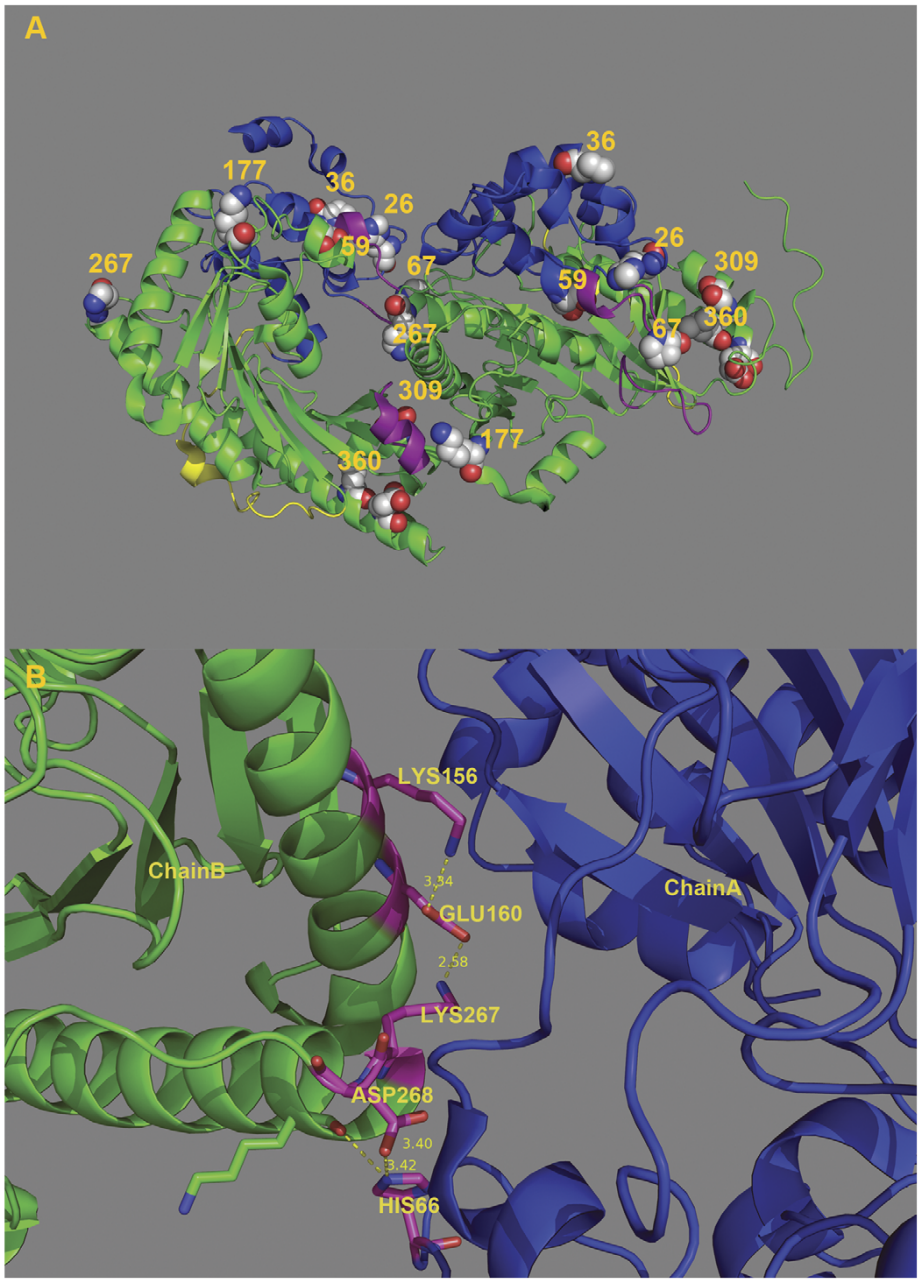
The distribution of the 9 SNP mutations on the CKB protein (A) and location of site 267 at the interface of dimerization (B). The NTD (blue), linker (yellow) and CTD (green) of CK are shown. Two catalytic loops (60–70, 323–332) are purple. Pictures were produced by the PyMOL program.

However, there are few reports of diseases that are directly caused by CKB deficiencies in humans to date. Nevertheless, data from CKB knock-out mice suggest that CKB is important for habituation, spatial learning behavior and the determination of seizure susceptibility [22], [23]. Mice lacking CKB also show defective thermoregulation [24]. From the CKB (Protein ID: NP_001814.2) SNPs in NIH SNP database build 130, 2009, We chose eight based on the location of the SNP in the related structure (PDB Protein ID: 3DRE) and on the principles of a protein stability prediction [25]. Essentially, missense SNPs which are close to active site, close to dimer interface, or buried inside were selected. These SNPs are H26Y (rs11545352), P36T (rs11545350), T59I (rs11545344), P67Q (rs11545348), K177R (rs36002620), K267E (rs13558), S309L (rs35156510) and L360F (rs12505) (Fig. 1A). The biochemical and biophysical properties of these mutants and of wild-type CK were measured and compared to find possible mechanisms for potential diseases induced by CK dysfunction caused by SNP disturbance.

## Materials and Methods

### Materials

ATP and Tris were Amresco products. Thymol blue, ultra-pure guanidine hydrochloride (GdnHCl), 1-anilinonaphthalene-8-sulfonate (ANS) and isopropyl-1-thio-β-d-galactopyranaside (IPTG) were purchased from Sigma. All restriction enzymes were obtained from Takara. The DNA ligation kit (named Ligation High) is a product of TOYOBO from Japan. Other reagents were local analytical grade products.

**Table 1:** Enzymatic spectra and stability parameters of WT and mutant CKs.

CKs	*k*_m_-creatine (mM)	*k*_m_-ATP (mM)	*k*_cat_ (S^−1^)	*k*_cat_/*k*_m_ -ATP (M^−1^S^−1^)	ANS intensity	*E*_max_ (nm)	*T*_m_ (°C)
WT	3.6±0.7	0.39±0.08	148±6	0.38±0.02	44.6±1.9	333	42.7±0.7
H26Y	4.5±1.2	0.89±0.08	**45±1**	**0.051±0.001**	**90.1±0.9**	**335**	**34.9±1.0**
P36T	3.9±0.1	0.2±0.06	**49±5**	**0.25±0.03**	**69.1±3.0**	**334**	**32.3±0.5**
T59I	**24.4±4.3**	**3.8±0.6**	**12±1**	**0.0032±0.0003**	**69.7±3.9**	**334**	44.7±0.6
P67Q	8.1±0.7	0.40±0.08	114±6	0.29±0.02	55.6±1.7	333	40.7±1.0
K177R	3.9±0.2	0.33±0.07	111±8	0.34±0.02	63.8±3.0	333	41.6±1.3
K267E	3.8±0.3	0.47±0.05	**192±12**	0.41±0.03	53.5±1.1	333	**38.0±0.9**
S309L	4.8±0.4	0.39±0.09	143±7	0.37±0.02	49.4±0.4	333	42.3±0.4
L360F	4.7±0.2	0.42±0.1	130±6	0.31±0.01	44.7±0.8	333	40.9±0.7

Data are presented as the mean ± sd (n = 3). Abnormal values are in bold.

### Protein Expression and Purification

The wild-type (WT) human brain-type creatine kinase (CKB) gene was amplified according to the published sequence (GenBank accession number NP001814) from the total cDNA of the HeLa cell [26]. SNP mutants were generated from the wild-type gene on a pMD-18T vector (Takara) using the DpnI method. See supplemental data for the primer sequences (Method S1).

All of the constructed genes were then ligated into a pET-21b expression vector. The WT and mutant CKs were expressed in Escherichia coli BL21 [DE3]-pLysS (Stratagene, Heidelberg, Germany). The protein expression and purification procedures are described in a previous work [26] with some changes. The *E. coli* cells were first cultured at 37°C to reach an OD_600_ between 0.6–0.8, and then 0.4 mM of IPTG was used to induce the cells. After incubating for approximately 18 h at 20°C, the culture was harvested and stored at –80°C. The cells were resuspended in 5 mM Tris-HCl, pH 8.0. After centrifugation at 13,000 rpm for 1 h, the supernatant of the cell lysate was loaded onto a DEAE Sepharose Fast Flow anion-exchange column (GE). Elution of the enzymes was carried out on a linear NaCl gradient from 0 to 0.6 M, and then the protein was concentrated and loaded onto a Hiload^TM^16/60 Superdex 200 prep grade column (GE). The enzymes were dissolved in 5 mM Tris-HCl buffer (pH 8.0) and stored at –20°C. The proteins were over 95% pure, based on SDS-PAGE analysis. The protein concentration was determined using a Bradford assay [27]. Protein oligomerization states were checked by native-PAGE with a 3.75% stacking gel and an 8% separating gel. Electrophoresis was carried out with Bio-Rad Mini-PROTEIN equipment on an ice bath. The buffer for electrophoresis was pre-cooled on ice. The gel was stained after electrophoresis with Coomassie blue R-250 and photographed.

**Figure 2:**
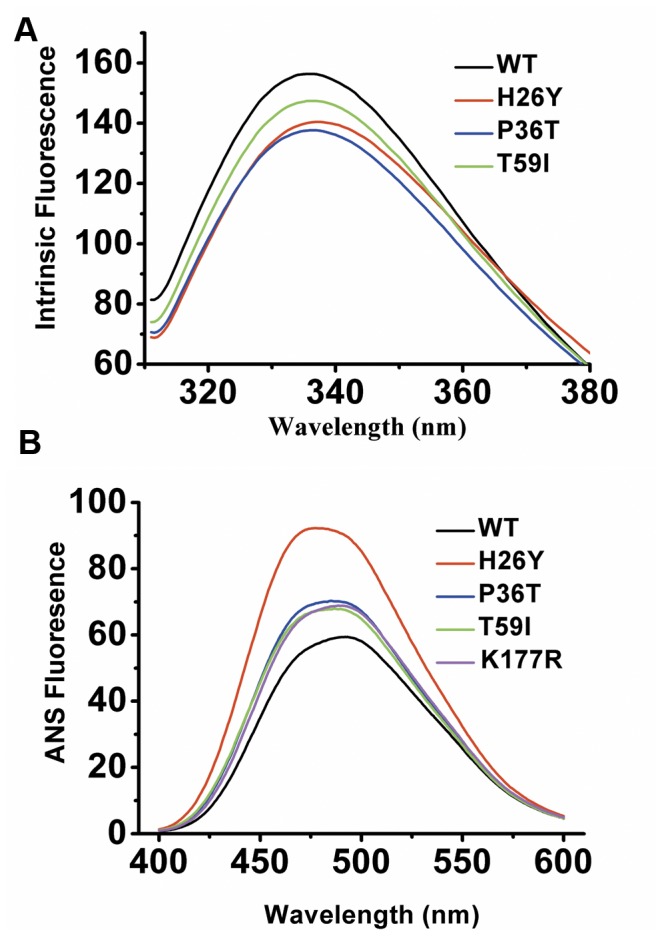
Effect of the SNP mutations on the enzyme structure: Trp (A) and ANS (B) Fluorescence spectra. CKs were dissolved in 20 mM Tris-HCl buffer, pH 8.0, with a final concentration of 0.2 mg/ml. Intrinsic fluorescence spectra were recorded using an excitation wavelength of 295 nm and an emission wavelength range of 300–400 nm. ANS fluorescence spectra were obtained by adding a 60-fold molar excess of ANS to the samples, which were then incubated for 30 min in the dark and then recorded from 400–600 nm. All experiments were carried out at 25°C and repeated at least three times. The baselines of both spectra were subtracted. Only the mutants that varied from WT CK are displayed.

**Figure 3:**
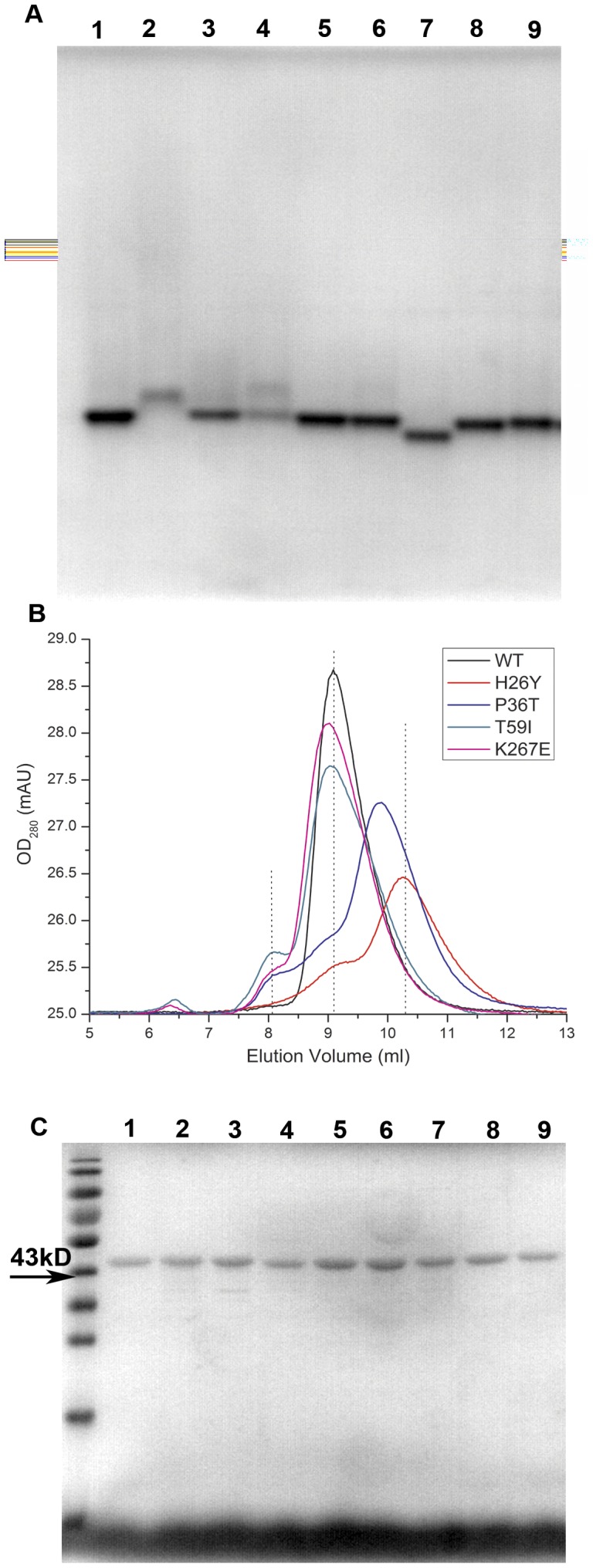
Stability of CKB SNP mutations. (A) The native polyacrylamide gel electrophoresis analysis (native-PAGE) of WT and mutant CKs. Lanes 1–9 represent WT, H26Y, P36T, T59I, P67Q, K177R, K267E, S309L and I360F, respectively. The protein concentration of the loaded samples was 2 µg. The conditions used for electrophoresis can be found in the methods section. (B) Gel filtration of WT, H26Y, P36T, T59I, and K267E SNPs. (C) SDS-PAGE of WT, H26Y, P36T, T59I, P67Q, K177R, K267E, S309L and I360F(Lanes 1–9).

### Enzyme Kinetics

CK activity was determined using the pH-colorimetry method [28]. The starting pH was 8.5. Both *V*_max_ values and *k*_m_ values of ATP and creatine were obtained by fitting the data to the Michaelis-Menten equation. All experiments were carried out on a Thermo Helios Gamma UV-VIS spectrophotometer.

### Thermal Stability and Reactivation of Wild-type and Mutant CKs

Wild-type and mutant CKs were diluted to a final concentration of 0.2 mg/ml with 5 mM Tris-HCl buffer, pH 8.0. Enzymes were incubated at given temperatures varying from 25°C to 60°C for 10 min; then, activities were immediately determined. To reduce the errors caused by the low activity of some mutants, a relatively higher protein concentration was used in these cases. The experiments were repeated at least three times. In the reactivation experiments, CKs were first incubated at 45°C for 30 min and then were recovered on ice overnight.

**Figure 4:**
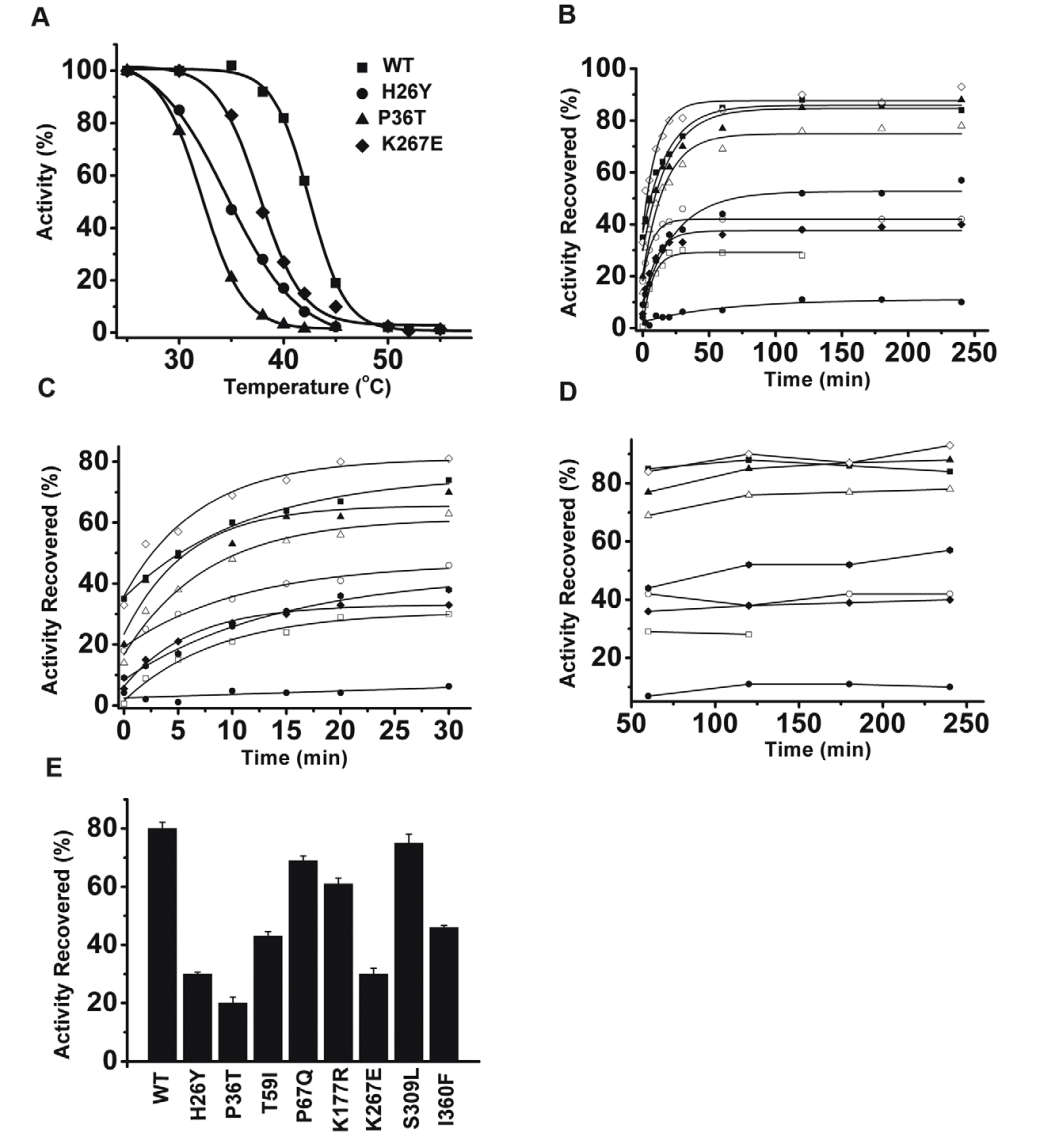
Thermal stability and thermal reactivation of CKB SNP mutations. (A) Thermal stability of WT and mutant CKs: WT (square), H26Y (circle), P36T (triangle) and K267E (diamond). The protein concentration of WT, P36T and K264E was 0.2 mg/ml. A relatively higher concentration (7.3 µmol) was used for H26Y. After being heated at the given temperatures for 10 min, the activity was immediately determined. The activity of the native enzymes at 25°C was assumed to be 100%. (B) Thermal reactivation time course of all enzymes: WT (filled square), H26Y (open square), P36T (filled circle), T59I (open circle), P67Q (filled triangle), K177R (open triangle), K267E (filled diamond), S309L (open diamond) and I360F (filled regular hexagon). Samples (0.2 mg/ml) were first heated at 45°C for 30 min and then cooled at 25°C. Activities at different time intervals were determined. Activities of untreated samples were assumed to be 100%. (C) and (D) are the enlarged part of the thermal reactivation time course. (E) The reactivation ability against thermal inactivation of WT and mutant CKs. Samples were prepared as described above. Reactivation of CKs was performed on ice overnight. Data are presented as the mean ± sd (n = 3).

### Intrinsic and ANS Fluorescence Measurement

Intrinsic and ANS fluorescence measurements were carried out on a Hitachi F-2500 fluorescence spectrophotometer with a 1 cm path-length cuvette. The protein intrinsic fluorescence had an excitation of 280 nm or 295 nm and was recorded from 300 nm to 400 nm. To determine the ANS fluorescence, each sample was first incubated with a 60-fold molar ratio of ANS for 30 min in the dark at 25°C and was then excited at 380 nm and recorded from 400 nm to 600 nm.

### Gel Filtration

Proteins (100 µl of 0.1 mg/ml) were loaded on a Superdex 200 10/300 GL column (GE Healthcare) and OD280 was monitored. The running buffer contains 5 mM Tris, pH 8.0. In the denatured gel filtration experiments, proteins were pre-denatured overnight on ice in GdnHCl. The running buffer contains the same concentration of GdnHCl as the denatured protein sample. Other experimental conditions are the same as in the native Gel filtration.

**Figure 5:**
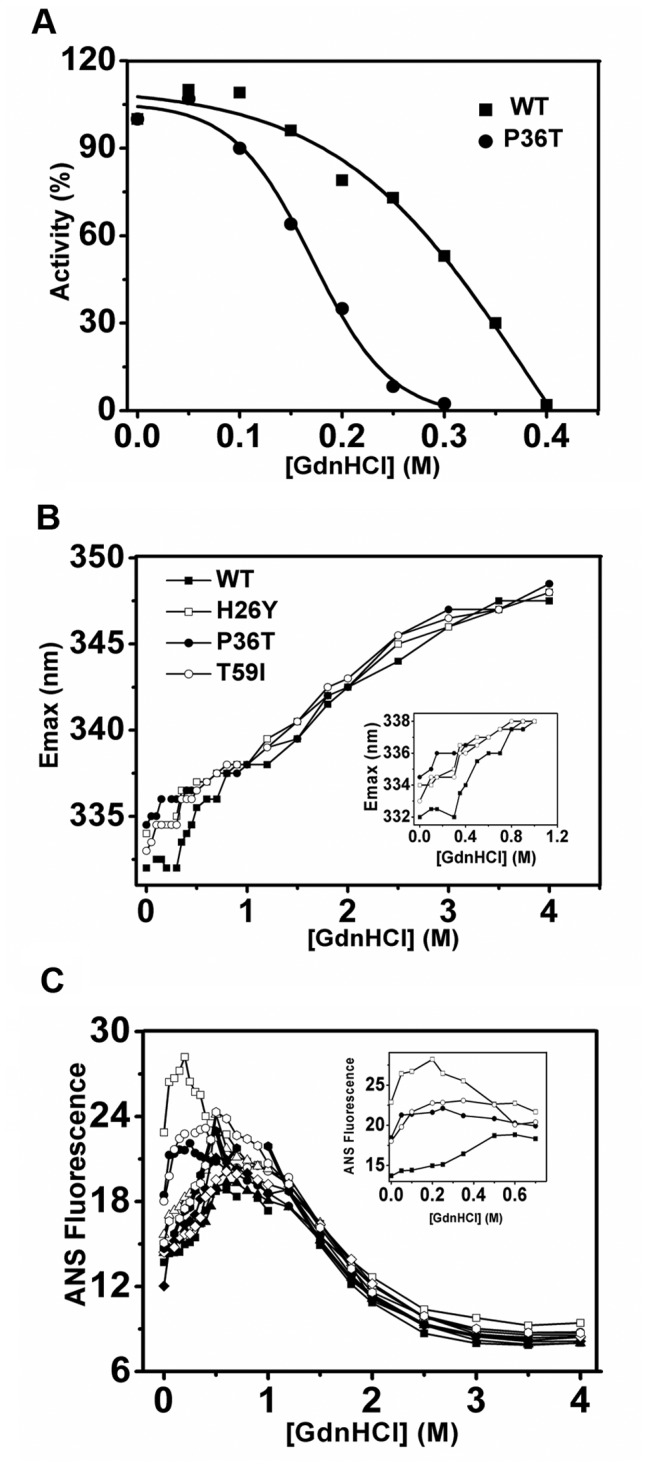
Unfolding of WT and mutant CKs in GdnHCl solution. The activities at different concentrations of GdnHCl are plotted in (A). The transition of the proteins was monitored by both the maximum emission wavelength (Emax) of intrinsic fluorescence (B) (excited at 280 nm) and the intensity of ANS fluorescence at 470 nm (C) (excited at 380 nm). The protein concentration was 0.1 mg/ml. Other details of the spectroscopic experiments were the same as those in Fig. 2. Labels of different data series were the same as in Fig. 4. Inserts are enlarged views of the curves at low GdnHCl concentrations.

### Equilibrium in GdnHCl Solution and Phase Diagram Analysis

Proteins (0.1 mg/ml) were incubated overnight at 25°C in 50 mM Tris-HCl buffer (pH 8.0) that contained various concentrations of GdnHCl; the reaction proceeded until no further change in the conformation parameters was observed. Fluorescence data were analyzed according to the phase diagram method, which is sensitive for detecting any intermediate state [29], [30]. The essential point of this method is to build up a diagram of I_λ1_ versus I_λ2_, where the I_λ1_ and I_λ2_ are the fluorescence intensities at two different emission wavelengths. The total fluorescence intensity (I_λ_) is described as a two-component system:
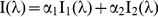
(1)

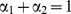
where α_1_ and α_2_ are the relative content of I_λ1_ and I_λ2_, respectively. Substituting α_1_ (α_1_ = 1 - α_2_), Eq. 1 could be written in the format below:



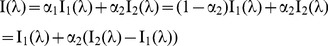
(2)Then, α_2_ can be determined by detecting the fluorescence intensities at two different wavelengths: λ_1_ and λ_2_. Two equations can then be obtained:
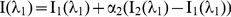
(3)

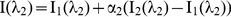
(4)

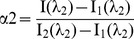
(5)


This configuration allows for the determination of the relationship between I(λ_1_) and I(λ_2_) by substituting α2 from Eq. 3 into Eq. 5:
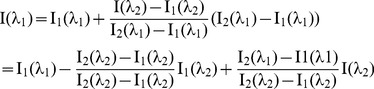
(6)


The following assignments are made:
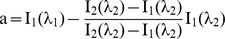


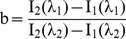



Eq. 6 can be written as follows:
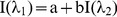
(7)


When applied to protein folding, if changes in the protein environment lead to the all-or-none transition between two different conformations, Eq. 7 predicts that the dependence I(λ_1_)  =  f(I(λ_2_)) will be linear. The non-linearity of this function reflects the sequential structural transformations. Moreover, each linear portion of the I(λ_1_)  =  f(I(λ_2_)) dependence describes an individual all-or-none transition. In practice, only wavelengths with different slopes of the spectrum will be informative, such as 320 and 365 nm. The fluorescence data were normalized to I320 and I365 from the spectra of the fully unfolded samples.

**Figure 6:**
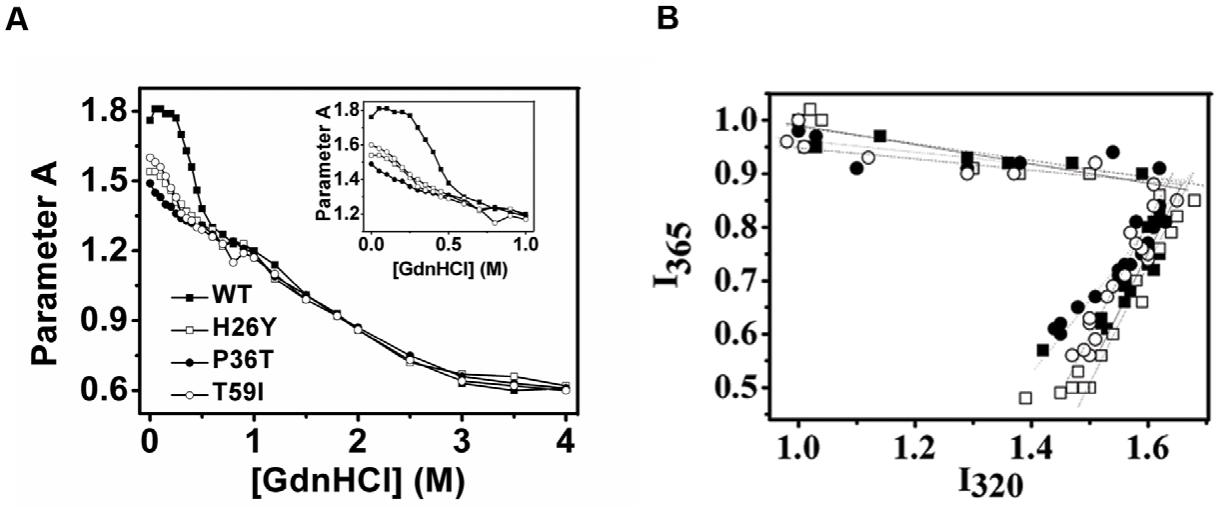
Parameter A and phase diagram analyses of intrinsic fluorescence data. (A) Parameter A was produced by dividing the fluorescence intensity at 320 nm (I_320_) by the intensity at 365 nm (I_365_). (B) In the phase diagram analysis, the fluorescence data were normalized by the I_320_ and I_365_ of samples fully denatured in 4 M GdnHCl. The data used was from Fig. 3. Only WT CK (filled square), H26Y (open square), P36T (filled circle) and T59I (open circle) are shown in (B); other mutants are the same as WT CK. Inset are enlargements of Parameter A at low GdnHCl concentrations.

## Results

### CK Activity and Structure

The primary sequence of a protein determines its three-dimensional structure and thus its functionality. Changing an amino acid sequence often leads to aberrant protein folding. To identify whether our SNP mutations in CKB affect the catalytic properties of the enzyme, both the enzymatic activity and the kinetic constants of these CKB SNPs were measured. Among all eight mutants, H26Y, P36T, T59I, and K267E have disrupted catalytic efficiency. K267E has increased enzymatic activity - approximately 30% higher than that of wild-type (Table 1). The remaining activity of H26Y, P36T and T59I is 30%, 33% and 8.1%, respectively. The binding affinity of the T59I mutant for substrates decreased about 8-fold for both creatine and ATP, as was reflected by the much-increased *k*_m_ values. Because Thr59 does not directly participate in substrate binding, the decreased substrate binding affinity indicates a global structure perturbation that remotely affects the structure of the binding site. Interestingly, the other two mutants, H26Y and P36T, which have unaffected *k*_m_s, also showed much lower enzymatic activity. These two sites are located at the very beginning of the N-terminal domain (NTD) of CK, which is far from the substrate binding pocket. Consequently, it is not surprising that these two SNP mutants possess native binding behavior. The much-decreased enzymatic activity may again be attributed to a perturbed global structure, which may also lead to decreased thermal stability. Notably, the structure formed by aa 26 - aa 36 was close to the catalytic loop (aa 60– aa 70) in the native structure, so a perturbation in this region may also affect the movement of the catalytic loop (Fig. 1A), leading to perturbed catalytic behavior.

The intrinsic fluorescence and ANS fluorescence of the mutant CKs were measured to further decide whether these mutations caused structural changes relative to WT CK. The emission maxima of the intrinsic tryptophan fluorescence of H26Y, P36T and T59I shifted slightly; the intensity also dropped by 5–10% (Fig. 2A). This finding suggested that the Trp residues of these mutants were more exposed to solvent and that the structures of these mutants were slightly opened. The ANS fluorescence intensity of H26Y increased more than two-fold, while the ANS fluorescence intensity of both P36T and T59I increased by approximately 55% (Fig. 2B). The increased ANS fluorescence intensity indicated that there was internal hydrophobic structure exposed and reflected a global structural perturbation caused by the mutations. In fact, mutants with more hydrophobic exposure (H26Y, P36T and T59I) have wider molecular mass distributions (Fig. 3A) and are prone to dissociate into monomers and/or form low-order oligomers (Fig. 3B). All mutants display similar monomeric mass in SDS-PAGE (Fig. 3C). It is not surprising that T59I has a lower activity due to its low substrate binding affinity. P36T forms low-order oligomers, which leads to its low activity. These results suggest that the low activity of the H26Y, P36T and T59I mutants appears to result from different mechanisms.

### Thermal Stability and Reactivation of the CKs

The thermal stability of the SNP mutants and WT CK was evaluated by the residual activity after a 10 min heat treatment (Fig. 4A). Enzymes were incubated at given temperatures, and the residual activity was immediately determined at 25°C. The thermal stability of the H26Y, P36T and K267E mutants decreased; in particular, the T_m_s of H26Y and P36T dropped below the physiological temperature of 37°C. The T_m_ value of H26Y is 34.9±1.0°C, and this mutant completely lost its activity after ten minutes of treatment at approximately 45°C. P36T had a much lower T_m_ value of 32.3±0.5°C, and this mutant completely lost its activity after 10 minutes heating at 40°C. Interestingly, the K267E mutant, which has a relatively high activity, is less thermally stable, with a T_m_ value of approximately 38.0±0.9°C. The kinetics of the reactivation process of WT and mutant CKs were monitored. Only P67Q and S309L could regain an activity level that was comparable to that of WT CK (Fig. 4B-4E).

### Effect of SNP Mutants on CK Folding

Single amino acid mutations often lead to the aberrant folding of a protein and may cause serious diseases, such as sickle-cell anemia. We monitored the unfolding of the WT and SNP mutant proteins in GdnHCl solution by spectroscopic methods. The D_1/2_ (concentration of the denaturant at 50% remaining enzymatic activity) of most of the variants is approximately 0.31 M, while that of P36T dropped to 0.17 M (Fig. 5A). According to Tsou’s theory [31], the active site is more sensitive to GdnHCl-induced inactivation compared to the overall enzyme structure. A reasonable explanation for the susceptibility of P36T to GdnHCl is that the CK dimers dissociated into monomers (Fig. 3B and Figure S1). As shown in Fig. 5B, the maximum emission wavelength of the intrinsic fluorescence (Emax) shifted to the red from 331.5 to 337 nm when the GdnHCl concentration increased from zero to approximately 0.6 M (Fig. 5B). The ANS fluorescence intensity of the WT CK and most SNP mutants reached a maximum approximately 0.6 M GdnHCl, although the maxima for H26Y and P36T came at the much lower GdnHCl concentration of approximately 0.3 M. The starting intensities of both mutants were also stronger compared to that of WT CK (Fig. 5C). Gel filtration experiments also indicate that H26Y and P36T are more susceptible at GdnHCl concentrations of 0–0.3 M (Figure S1). These results indicate that the sequence at the NTD is very important for the correct folding of CK. Two different mutations here both greatly affected the CK resistance to GdnHCl. The folding of CKB is very similar to the folding pattern of HMCK [32], [33], which is a three-state dominated process involving a molten globular intermediate (N←→MG←→U). The difference between the WT CK and the H26Y, P36T and T59I mutants was only detected at low denaturant concentrations, implicating a similar mechanism at the later stage of the chaotic reagent-induced unfolding process.

We then used parameter A and phase diagram analyses to characterize the folding process of WT CK and the SNP mutants. Both are sensitive tools for identifying folding intermediates [34]. As presented in Fig. 6A, parameter A, which indicates the shape of a molecule, is identical under high GdnHCl concentration (above 0.6 M) for all of the proteins tested here. By contrast, the H26Y, P36T and T59I mutant parameter A curves split from the curves of WT CK and the other mutants at low GdnHCl concentrations. Furthermore, the phase diagram analysis indicates that all the transitions could be joined by two independent linear parts, which suggests that the folding pathway of all of the proteins is a three-state dominated process that includes one intermediate state. The joint position of two linear lines was also at the same GdnHCl concentration (approximately 0.6 M). These results agreed with those from the direct spectra analysis in Fig. 3, indicating that not all of the mutations affected the folding pathway of CK. The disparity between WT and mutant CKs unfolding at low detergent concentrations was more likely due to differences in the native structures rather than interference in the folding because of the mutations.

## Discussion

CKB is mainly distributed in neural tissues and is found to be associated with the neuron-specific K^+^-Cl^−^ co-transporter KCC2 [14], which indicates that CK may participate in neural transduction. In fact, mice lacking CKB show abnormal behavior, which is a reflection of the coordination of neural system. Genetic mutations of CK would thus be crucial for diseases caused by CK dysfunction. Among all our SNP mutants, only H26Y, P36T, T59I and K267E have disrupted enzymatic activities. Three out of four mutants have relatively lower activities compared to those of WT CK, while K267E showed a higher catalytic efficiency. The low activity of SNP mutant enzymes might be one of the reasons for the decreased activity seen in clinical observations, thus giving evidence for a potential pathogenicity that might cause serious diseases. The H26Y and P36T mutants have similar binding kinetic constants to that of WT CK but possess only approximately 33% activity compared to WT CK. Note that both sites are located at the very beginning of the CK N-terminal domain, so they are far away from the catalytic pocket of the enzyme [35]. It is most probable that these mutations did not affect the conformation of the active site, thus the substrate binding affinity was not disturbed. However, they are spatially close to one of the catalytic loops, so this structural alternation may affect the conformation change of the catalytic loop during catalytic action, thus leading to decreased enzymatic activity. Thr59 did not directly participate in substrate binding, so its lower binding affinity may be due to the structural changes caused by the residue replacement. Note that Thr59 is located at the very beginning of one catalytic loop (residues 60–70), so the low activity of this mutant might also be caused by disordered loop movement. Although site Thr59 was near the Asp54 site that forms a hydrogen bond with R148 [35], [36], the mutation did not have any effect on the thermal stability of the protein. While a previous report elucidated that a D54G mutant had a wide M_W_ distribution due a the fast equilibrium between the dimeric and monomeric forms, the catalytic properties, structure and stability were disrupted [37]. The H26Y and P36T mutants have lower T_m_ values than the WT CK (Table 1). Meanwhile, the reactivation fractions of heat-treated H26Y and P36T mutants were extremely low (approximately 30% and 20%, respectively). Because CKB and the SNP mutants were not prone to form large aggregates (data not shown) during thermal treatment (no change in turbidity), the inactivated states of the proteins must include the soluble aggregate because the reactivation productions were so low for the H26Y and P36T mutants. The tendency to form soluble aggregates should be due to hydrophobic exposure caused by the genetic mutations (Fig. 2B). In fact, almost all of the mutations, except for P67Q, K177R and S309L, have decreased reactivation abilities (Fig. 4B to E), which may be the effect of structural alternations that are not detectable by our current techniques. Because CKB was mainly distributed in the nervous system, SNP mutants with disrupted thermal reactivation abilities may not be favorable in the event of body temperature fluctuations, i.e., fevers. Because P36T almost lost activity and H26Y and K267E have about half activity under physiological temperature (Fig. 4A), if they also retain the same property *in vivo*, the individual would suffer from genetic problems, as elucidated by CKB knockout mice [22], [23], [24].

In the thermal reactivation experiments, H26Y, P36T and T59I caused significant structural changes. While the structures of other mutants may also be disturbed, they are not detectable with our current techniques. The CTD is particularly important for the structural stability of CK [38]. Mutation of the 26^th^, 36^th^ and 59^th^ amino acids at the NTD results in an opened structure, as reflected by the intrinsic and ANS fluorescence spectra (Fig. 1A and Fig. 2). Previous reports also showed that D54G mutation of CK has a similar effect on the CK structure [37]. Lys267 is shown to be located at the dimerization interface within the CKB dimer (Fig. 1B). Although the enzymology constants were similar and the structure was identical to that of WT CK, the mutation of Lys267 to Glu greatly disturbed the thermal stability and mobility of the native protein (Fig. 3A). Although Lys267 is not a key residue in the dimerization of CK subunits, a mutation here does affect dimerization by affecting the overall protein structure. The folding of WT CK and the mutants is a three-state process involving a MG intermediate, as shown by spectra methods (Fig. 5 and Fig. 6). This process is very similar to the folding process of rabbit muscle CK and other derived proteins [32], [37], [39]. The similar position of the maximum ANS fluorescence intensity indicates that most mutants have the same MG state distribution, except H26Y, which exhibits an earlier peak and a higher starting intensity. One possibility for this abnormality is that the H26Y replacement disrupted the structure of the enzyme, leading to extensive hydrophobic interface exposure (Fig. 5C). The difference between WT and mutant CK was the transition from the MG intermediate to the native state. The partially folded state of the mutants made it easier for them to form soluble aggregates, as indicated by the thermal reactivation assay (Fig. 4).

In conclusion, SNPs are changes in genomic DNA that often lead to the alternation of protein sequences. The mutations may affect the enzymatic activity, protein stability and folding pathways of the protein. The mutant proteins may have higher energies than those of the wild type, and they may be more prone to form off-pathway aggregates [40], [41]. This observation was particularly true for CKB SNP mutants because the H26Y and P36T mutants were found to be in a partially folded state and were more prone to form soluble aggregates during heat stress. They also have a lower degree of thermal reactivation recovery. The low activity of H26Y and T59I and the instability of H26Y, P36T and K267E, even at physiological temperature, suggests possible mechanisms for diseases caused by CK deficiency, which has been reported in the literature [42].

## Supporting Information

Figure S1 H26Y and P36T are more susceptible to low concentrations of GdnHCl. Gel filtration of WT, H26Y and P36T at GdnHCl concentrations of 0–0.3 M. The elution profiles of WT (A), H26Y (B) and P36T (C), in GdnHCl concentrations as indicated.
(TIF)

Method S1 Primer sequences for generating the SNP mutants.
(DOCX)
