# Supplementary figures and images for: Effect of SNPs on Creatine Kinase Structure and Function: Identifying Potential Molecular Mechanisms for Possible Creatine Kinase Deficiency Diseases
Source: PLoS One. 2012 Sep 25;7(9):e45949. doi: 10.1371/journal.pone.0045949 (PMC3457962; doi:10.1371/journal.pone.0045949)

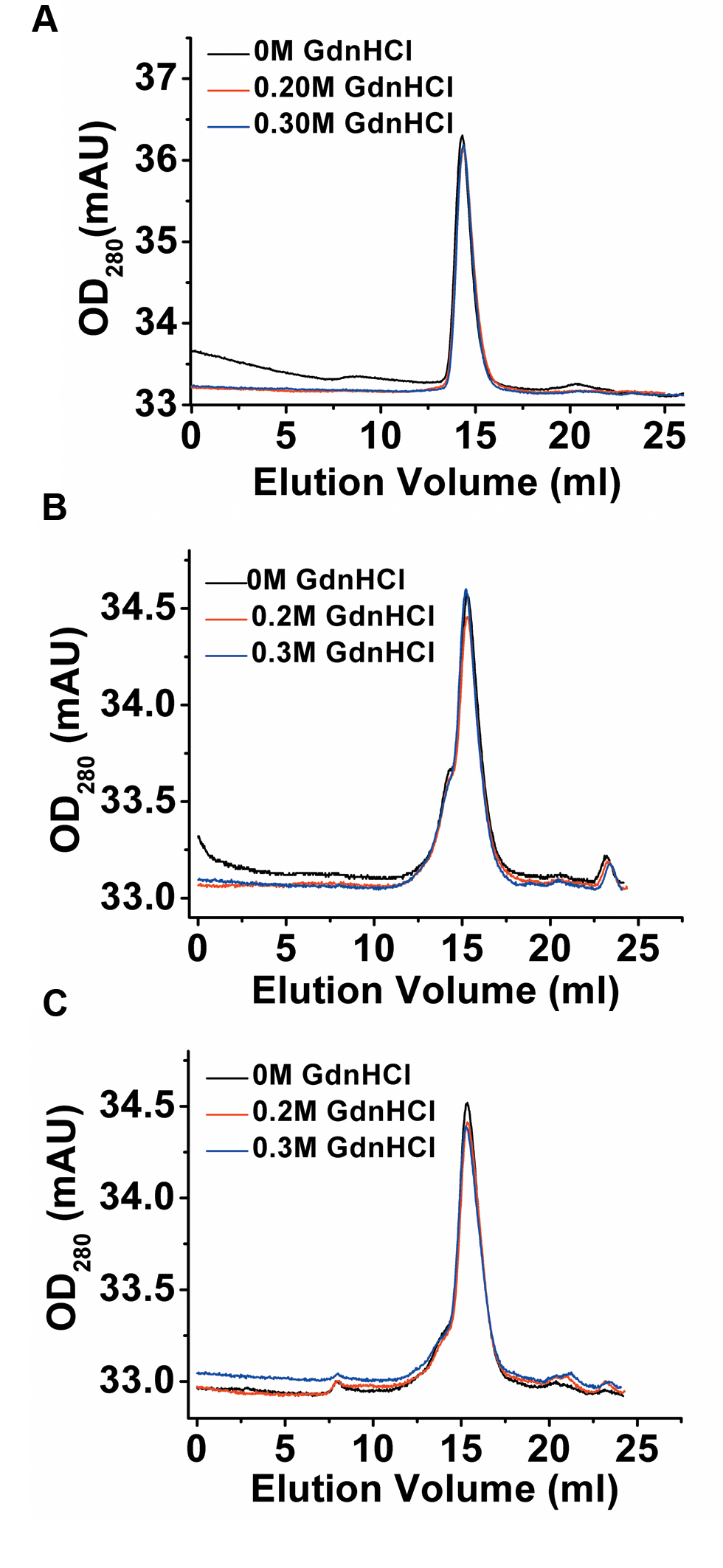

Supplement: Figure S1 — H26Y and P36T are more susceptible to low concentrations of GdnHCl. Gel filtration of WT, H26Y and P36T at GdnHCl concentrations of 0–0.3 M. The elution profiles of WT (A), H26Y (B) and P36T (C), in GdnHCl concentrations as indicated. (TIF) [file pone.0045949.s001.tif]
