# Supplementary material for: Effect of SNPs on Creatine Kinase Structure and Function: Identifying Potential Molecular Mechanisms for Possible Creatine Kinase Deficiency Diseases
Source: PLoS One. 2012 Sep 25;7(9):e45949. doi: 10.1371/journal.pone.0045949 (PMC3457962; doi:10.1371/journal.pone.0045949)
Supplement: Method S1 — Primer sequences for generating the SNP mutants. (DOCX) [file pone.0045949.s002.docx]

**Supplemental method**

**Primer sequences of creatine kinase SNP mutants:**

H26Y-For, 5’-CCTGAGCGCCTACAACAACCAC-3’;

H26Y-Rev, 5’-CATGTGGTTGTTGTAGGCGCTC-3’;

P36T-For, 5’–TGCTGACCACCGAGC TGTACGC-3’;

P36T-Rev, 5’-TACAGCTCGGTGGTCAGCACCT-3’;

T59I-For 5’-ACGTCATCCAGATAGGCGTGGA-3’;

T59I–Rev, 5’-TGTCCACGCCTATCTG GATGAC-3’;

P67Q-For, 5’-CCACCAGTACATCATGACCGTG-3’;

P67Q-Rev, 5’-CG GTCATGATGTACTGGTGGCC-3’;

K177R-For, 5’-CGCTCAGGAGCATG ACGGAGGC-3’;

K177R-Rev, 5’-TCCGTCATGCTCCTGAGCGCGT-3’;

K267E -For, 5’-TCAAGTCTGAGGACTATGAGTT-3’;

K267E-Rev, 5’-TCATAGTCCTCAGACTTGAAGA-3’;

S309L-For, 5’-ATGAGAAGTTCTTGGAGGTGCT-3’;

S309L-Rev, 5’-TTAAGCACCTCCAAGAACTTCT-3’;

L360F-For, TGAAGCTGTTCATCGAGATGGA-3’;

L360F-Rev, 5’-ATCTCGATGAACAGCTTCACTC-3’;
